# Bloqueio Divisional Anterossuperior Esquerdo Associado a Bloqueio Atrioventricular Induzido ao Teste Ergométrico

**DOI:** 10.36660/abc.20230200

**Published:** 2023-11-13

**Authors:** Andréa Marinho Falcão, Vagner L. Suares, William A. Chalela

**Affiliations:** 1 Hospital das Clínicas Faculdade de Medicina Universidade de São Paulo São Paulo SP Brasil Instituto do Coração do Hospital das Clínicas da Faculdade de Medicina da Universidade de São Paulo , São Paulo , SP – Brasil

**Keywords:** Teste de Esforço, Doença do Sistema de Condução Cardíaco, Doença da artéria coronariana

## Introdução

O teste de esforço (TE) é indicado em uma ampla variedade de situações clínicas. ^1^ Apesar das evidências consistentes do significado clínico das anormalidades de ST-T durante o exercício, pouca atenção tem sido dada ao desenvolvimento de distúrbios de condução intraventricular induzidas pelo exercício.

Os distúrbios de condução induzidos ao TE são incomuns e os bloqueios divisionais são ainda mais raros. Estima-se que bloqueios de ramo esquerdo (BRE) induzidos ao esforço apresentem uma prevalência que varia de 0,3%-0,5% e estão associados a prognóstico mais adverso (maior prevalência de doença coronária obstrutiva e insuficiência cardíaca). ^2^

Já os bloqueios divisionais (anterossuperior, anteromedial e posteroinferior) induzidos ao esforço se restringem a publicações de relatos de caso da literatura. ^3 , 4^ Essas alterações, quando presentes, elevam o risco de eventos cardiovasculares, sendo preditoras de pior prognóstico. Em geral, estão associadas a lesões críticas da artéria descendente anterior (DA) ou de tronco da coronária esquerda, já que o maior suprimento sanguíneo do ramo esquerdo do feixe de His e seus fascículos é da DA. ^4^

## Descrição

Trata-se de paciente de 77 anos, sexo feminino, encaminhada para consulta cardiológica. Apresentava história de dispneia, dor torácica e tontura com início há seis meses. Não fazia uso de medicações cardiovasculares e não apresentava fatores de risco cardiovascular. Apresentava-se ao exame físico em bom estado geral, frequência cardíaca (FC) de 64 bpm, pressão arterial (PA) 140x80 mmHg. A paciente estava alerta e orientada, eupneica, com pulsos simétricos nos quatro membros e sem edemas periféricos. Ausculta cardíaca tinha ritmo regular, bulhas normofonéticas e sem sopros, ausculta pulmonar normal. Demais aparelhos e sistemas sem alterações.

**Figura 1:**
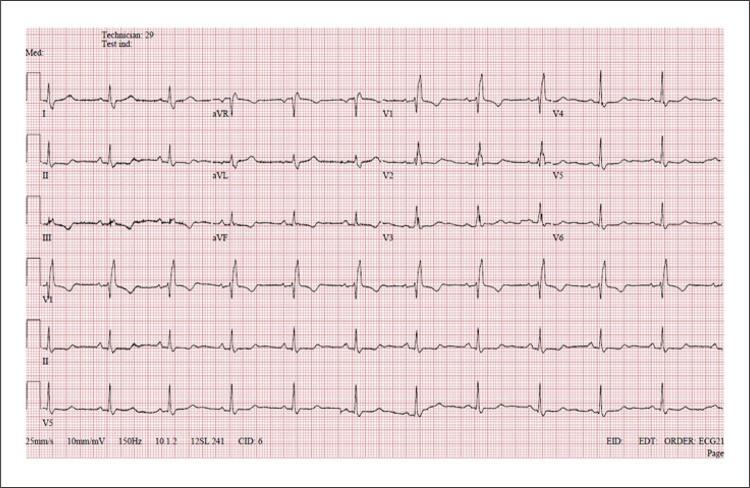
– Eletrocardiograma de repouso evidenciando BRD e alterações da repolarização ventricular.

O eletrocardiograma (ECG) de repouso evidenciou ritmo sinusal, FC de 60 bpm, BRD e alterações difusas da repolarização ventricular ( Figura 1 ). O ecocardiograma transtorácico evidenciou fração de ejeção ventricular esquerda (FEVE) de 61% (Teicholz), câmaras cardíacas de tamanhos normais não sendo observadas alterações na motilidade segmentar. Foram realizados cintilografia de perfusão miocárdica (CPM) associada ao TE com o protocolo Bruce e Holter-ECG de 24 horas.

**Figura 2:**
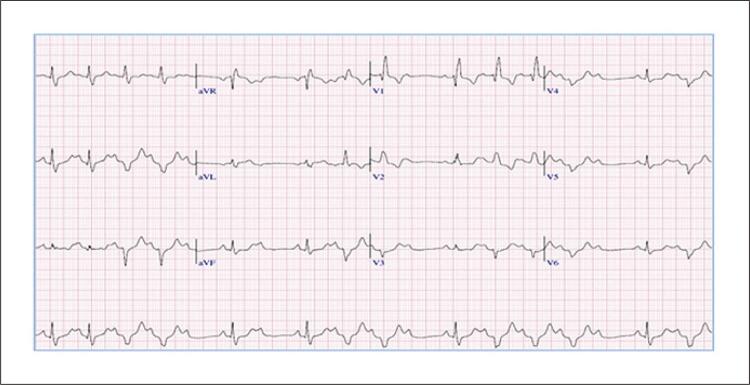
– Eletrocardiograma do esforço máximo, onde se evidencia BRD (padrão basal) alternado com complexos QRS com desvio acentuado do eixo à esquerda (BDASE) e acompanhado de BAV de 2º grau Mobitz II. FC aproximadamente de 100 bpm e manifestação clínica de pré-síncope.

**Figura 3:**
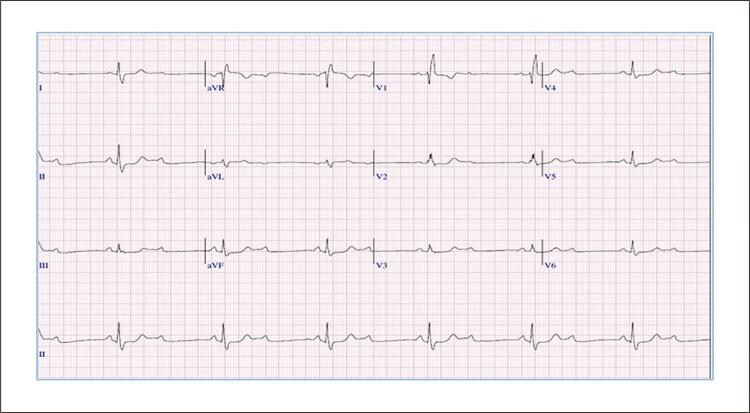
– Eletrocardiograma do 6º min da recuperação: BRD (padrão basal), desaparecimento do BDASE e BAV de 2º grau 2:1.

Ao final do 2º estágio do protocolo Bruce o BRD prévio associou-se a um BAV 2:1 e períodos de distúrbios de condução atrioventricular e intraventricular (BDASE) intermitente ( Figura 2 ), associado aos sintomas de fadiga extenuante, dor precordial e pré-síncope, que levaram à interrupção do exame. O teste durou 13 minutos 39 segundos, com pressão arterial (PA) variando de 122x63 mmHg a 202x68 mmHg no pico do esforço. A FC máxima alcançada foi de 97 bpm, correspondendo a 67% do previsto para a idade e carga de trabalho de 7 METs. Na fase de recuperação (6 minutos), retorno ao padrão basal de BRD associado ao BAV 2:1 ( Figura 3 ). A CPM não mostrou evidências de isquemia miocárdica induzida por esforço até alcançar a FC de 97 bpm.

Esses achados são compatíveis com doença do sistema de condução infra-nodal, devido ao aparecimento dos bloqueios, especialmente o BAV podem representar doença bilateral do sistema His-Purkinge. Além de muito raro, requerem intervenção rápida e adequada. O Holter de 24 horas evidenciou períodos de BAV 2:1 associado a bloqueio de ramo alternante nos períodos de maior FC. A paciente foi submetida ao implante de marca-passo atrioventricular permanente com sucesso, com melhora importante dos sintomas.

## Discussão

Os achados descritos representam um raro caso de bloqueio “trifascicular” ao esforço (BRD em repouso associado ao BDASE e BAV 2º grau Mobitz II ao esforço). O padrão eletrocardiográfico do distúrbio de condução BDASE é descrito na literatura. ^1 , 2 , 4 , 5^ Algumas vezes ele pode não ser reconhecido por profissional pouco treinado quando induzido ao TE. Seu diagnóstico requer atenção especial às mudanças do eixo cardíaco. ^6 , 7^ Os critérios para identificar o BDASE transitório ou permanente é o aparecimento de SÂQRS igual ou além de - 45º; rS em DII, DIII e aVF, com S3 > S2; QRS com duração < 120 ms; qR em D1 e aVL com tempo de início da deflexão intrinsecóide; (TIDI) > 45 ms ou qRs com “s” mínima em DI; qR em aVR com R empastado; diminuição de “ r “ de V1 até V3 e presença de s de V4 a V6. ^6 , 7^

No entanto, a presença de BDASE ao esforço também requer o diagnóstico diferencial com algumas situações como: atraso final de condução, outros distúrbios de condução do ramo esquerdo, arritmias ventriculares, hipertrofia ventricular esquerda, etc. ^7^ Apesar dos casos relatados na literatura de BDASE terem associação com DAC grave, isso não foi observado no nosso caso.

Já os BAVs induzidos durante o TE também são eventos bastante incomuns. A frequência relativa de aparecimento durante a fase de exercício do TE é reportada como 0,45%. ^8 - 11^ Na fase de recuperação, o tipo mais frequentemente relatado é o BAV de primeiro grau, com uma ocorrência variando entre 2,8% nos indivíduos abaixo dos 40 anos, até 11% naqueles acima de 60 anos. Não obstante sua raridade, o aparecimento de BAV no TE é de grande relevância na prática clínica, podendo auxiliar na definição de condutas em casos especiais. Entretanto, pouco se encontra publicado sobre o tema, mesmo em textos específicos sobre exercício físico e TE. Com base nesses pressupostos, temos que os principais mecanismos envolvidos no surgimento dos BAV durante o TE são os que seguem: desequilíbrio da regulação autonômica (regulação extrínseca); falha na regulação do sistema intrínseco nodal AV; degeneração do sistema de condução cardíaca; eventos isquêmicos comprometendo o nó AV, feixe de His ou ainda a parede inferior do VE - nesse último por promover ativação do reflexo vagal de Bezold-Jarish. ^12^ Assim, os casos encontrados na literatura de BAV induzido ao esforço ocorrem em pessoas idosas, mais de 60% tinham idade igual ou superior a 60 anos, sendo o indivíduo mais jovem de 31 anos, o que nos leva a supor a etiologia degenerativa como causa principal. O ECG de repouso, previamente ao aparecimento dos bloqueios, era normal em 40% dos casos e a alteração mais encontrada foi o BRD, como no caso descrito. A maioria dos BAV encontrados era de bloqueios de segundo grau 2:1 ou avançados (quase 90% dos casos). ^12^

É a localização do bloqueio no sistema de condução que normalmente determina o prognóstico no BAV de II grau. O BAV de 2º grau Mobitz I na grande maioria das vezes é de localização nodal e, portanto, responde ao sistema nervoso autônomo, melhora a condução AV ao exercício. Já nos BAV 2º grau Mobitz II, sua localização já é no sistema His-Purkinge e consequentemente não é influenciado pelo tônus adrenérgico do exercício.

No caso descrito, o BAV apresentado foi um bloqueio Mobitz II, de provável localização infra-nodal, mais comumente observado durante o esforço, já que o sistema His-Purkinje não é responsivo ao sistema nervoso autônomo. Estudos confirmam que nos pacientes em que se realizou o estudo eletrofisiológico invasivo (EEF), constatou-se que a localização dos bloqueios AV era infra-nodal em 85% deles. Tais achados, somados à presença de sintomas, justificam a indicação de implante de marca-passo definitivo em grande parte dos casos; ^7 , 13 - 15^ isquemia miocárdica raramente foi reportada como causa dos BAVs durante exercício. Mesmo em alguns casos em que se demonstrou a presença de obstrução coronariana, o tratamento da lesão obstrutiva não resolveu o BAV, sugerindo que outros mecanismos além da isquemia estavam presentes nesses indivíduos, semelhante ao caso apresentado, em que a perfusão miocárdica foi normal, mesmo em baixa FC alcançada. Assim, o TE com a sua análise multifatorial foi de fundamental importância. A avaliação da resposta dromotrópica expôs os BDASE não conhecidos previamente, associados à manifestação clínica de pré-síncope.

## Conclusão

O TE foi fundamental para o diagnóstico e manejo clínico do caso e seus achados foram suficientes para indicar o implante de um marca-passo atrioventricular permanente.
