# Supplementary material for: Bloqueio Divisional Anterossuperior Esquerdo Associado a Bloqueio Atrioventricular Induzido ao Teste Ergométrico
Source: Arq Bras Cardiol. 2023 Nov 13;120(11):e20230200. [Article in Portuguese] doi: 10.36660/abc.20230200 (PMC10697673; doi:10.36660/abc.20230200)
Supplement: Translation

# Translation: Left Anterior Fascicular Block Associated with Atrioventricular Block during Exercise Stress Test

**Authors:** Andréa Marinho Falcão, Vagner L. Suares, William A. Chalela

**Affiliations:** 1 Brazil Instituto do Coração do Hospital das Clínicas da Faculdade de Medicina da Universidade de São Paulo, São Paulo, SP – Brazil

**Keywords:** Exercise Test, Cardiac Conduction System Disease, Coronary Artery Disease

## Introduction

The exercise stress test (EST) has been indicated in various clinical situations. ^1^ Despite the robust evidence about the clinical significance of ST-T abnormalities during exercise, little attention has been paid to the development of exercise-induced intraventricular conduction disorders.

Conduction disorders and fascicular blocks induced during EST are rare. The prevalence of stress-induced left bundle-branch blocks (LBBB) ranges from 0.3%-0.5%, and frequently, the prognosis is more adverse (greater CAD and heart failure prevalence). ^2^

Data regarding stress-induced fascicular blocks (left anterior or posterior fascicular blocks) are restricted to case reports in the literature. ^3 , 4^ When present, these alterations increase the risk for cardiovascular events and are predictors of worse prognosis, usually associated with critical lesions of the left anterior (LAD) or left main coronary artery, given that most of the blood supply for the left bundle of His and its fascicles come from the LAD. ^4^

## Description

We describe a case of a 77-year-old female patient who was referred for cardiological screening. She presented with dyspnea CCS II/III, chest pain, and dizziness, which began six months before. She did not present any cardiovascular risk factors. Physical examination was normal: heart rate (HR) 64 bpm, blood pressure (BP) 140/80 mmHg. On physical examination, she was in good general condition, alert and oriented, eupneic, with symmetric pulses in the four limbs, and without peripheral edema. Normal heart sounds with regular rhythm and normal pulmonary auscultation. The other body parts and systems showed no alterations.

The resting electrocardiogram (ECG) showed sinus rhythm, HR 60 bpm, RBBB, and diffuse alterations of the ventricular repolarization ( Figure 1 ). A transthoracic echocardiogram showed a left ventricular ejection fraction (LVEF) of 61% (Teicholz), heart chambers of normal sizes, and no wall motion alterations. She also underwent myocardial perfusion scintigraphy (MPS) with an EST and Bruce protocol and 24-hour Holter ECG.

**Figure 1:** – Rest ECG - RBBB and alterations of the ventricular repolarization.

At the end of the Bruce protocol’s second stage, the previous RBBB pattern was associated with a 2:1 AV block and transient periods of light on the atrioventricular and intraventricular conduction disturbances (LAFB) ( Figure 2 ), associated with symptoms of strenuous fatigue, chest pain, and presyncope, which led to stop the exercise. The test lasted about 13 minutes, with BP ranging from 122/63 mmHg to 202/68 mmHg at peak exercise. The maximum HR achieved was 97 bpm, corresponding to 67% of the predicted for the age and the workload of 7 METs.

**Figure 2:** – Rest and peak exercise ECG: RBBB with QRS complex markedly deviated to the left (LAFB) in association with a second-degree Mobitz II AV block. HR nearly 100 bpm and clinical manifestation of presyncope.

During recovery (6 minutes), the baseline RBBB pattern was associated with the 2:1 AV block ( Figure 3 ). MPS showed no evidence of stress-induced myocardial ischemia until HR 97 bpm was achieved.

**Figure 3:** – ECG 6 min recovery: RBBB, disappearance of the LAFB, and 2:1 second-degree AV block.

The above findings are compatible with infra-nodal conduction system disease, as indicated by the emergence of blocks, particularly AV block, and they represent a bilateral His-Purkinje system disease. In addition to being very rare, they demand a quick and appropriate intervention. The 24-hour Holter revealed 2:1 AV block periods associated with alternating branch block during the periods of higher HR. The patient then underwent a permanent atrioventricular pacemaker implantation, with improvement of her symptoms.

## Discussion

The findings reported represent a rare case of “trifascicular block” on EST (RBBB at rest, associated with LAFB and second-degree Mobitz II AV block during exercise). The ECG pattern of the LAFB conduction disturbance is reported in the literature. ^1 , 2 , 4 , 5^ Sometimes, it may not be recognized by a less trained physician, especially when stress-induced. Its diagnosis demands special attention to the cardiac axis changes. ^6 , 7^ The criteria to identify a transient or permanent LAFB is the appearance of SÂQRS ≥- 45º; rS in DII, DIII and aVF, with S3 > S2; QRS < 120 ms of duration; qR in DI and aVL with a time of intrinsicoid deflection ≥ 50 ms or qRs with minimal “s” in DI; qR in aVR with notched R; “r” decreasing from V1 to V3, and presence of “s” from V4 to V6. ^6 , 7^

On the other hand, the presence of LAFB on effort also demands differential diagnosis with end-conduction delay, other left branch conduction disturbances, ventricular arrhythmias, left ventricular enlargement, etc. ^7^ Although the cases of LAFB in the literature have been associated with CAD, this was not observed in this case.

Atrioventricular blocks induced during EST are also very uncommon events. Their relative frequency during the EST is reported as 0.45%. ^8 - 11^ During the recovery phase, the most frequently reported type is the first-degree AV block, with an occurrence ratio ranging from 2.8% in individuals below 40 years of age to 11% in those older than 60. Despite their rarity, the appearance of an AV block during EST has great relevance in clinical practice and may help to establish the management in special cases. However, little is found in the literature about this theme, even in specific texts on EST. So, we may state that the mechanisms involved in the appearance of AV blocks during EST could be related to an imbalance of the autonomic regulation (extrinsic regulation), failure in the intrinsic AV nodal system regulation, degeneration of the cardiac conduction system, particularly in the older people; ischemic events impairing the AV node, His bundle or even the LV inferior wall – in the latter caused by promoting the activation of the Bezold-Jarish vagal reflex. ^12^ So, cases of stress-induced AV block in the literature occur in older people, more than 60% of them ≥ 60 y.o.; the youngest was 31 y.o., possibly having a degenerative etiology. In 40% of the cases, the rest-ECG before the appearance of blocks was normal, and the most common alteration was RBBB, similar to this case. Most AV blocks found were 2:1 second-degree or advanced blocks (almost 90% of the cases). ^12^

The site of the block along the conduction system normally determines the prognosis in the second-degree AV block. Most are Mobitz I AV block and localized in the AV node. Therefore, they respond to the autonomic nervous system and improve the conduction through the AV node during exercise. Regarding the second-degree Mobitz II AV blocks, their localization is already in the His-Purkinje system; consequently, they are not influenced by the adrenergic tone of the exercise.

The AV block described here was a Mobitz II, probably of infra-nodal localization, more commonly noticed during the effort since the His-Purkinje system is not responsive to the autonomic nervous system. Reports in patients invasively studied (by an invasive electrophysiologic study [EEF]) found an infra-nodal localization of AV blocks in 85% of them. Those findings and the presence of symptoms warrant the indication for definitive pacemaker implantation in most cases, ^7 , 13 - 15^ and myocardial ischemia has rarely been reported to cause AV blocks during exercise. Even in some cases where the presence of CAD was demonstrated, treating the obstructive lesion did not resolve the AV block, indicating that mechanisms other than ischemia might have been in action in those individuals. This is similar to the case presented, which had a normal myocardial perfusion result, even with the low HR achieved. Therefore, the multifactorial analysis of the EST was of crucial significance. The evaluation of the dromotropic response shed LAFB that had not been previously noticed in association with the clinical manifestation of presyncope.

## Conclusion

The EST was imperative for this patient’s diagnosis and clinical management, and these findings were enough to indicate a permanent atrioventricular pacemaker implantation.
